# Molecular Dynamics Simulation of the Influenza A(H3N2) Hemagglutinin Trimer Reveals the Structural Basis for Adaptive Evolution of the Recent Epidemic Clade 3C.2a

**DOI:** 10.3389/fmicb.2017.00584

**Published:** 2017-04-10

**Authors:** Masaru Yokoyama, Seiichiro Fujisaki, Masayuki Shirakura, Shinji Watanabe, Takato Odagiri, Kimito Ito, Hironori Sato

**Affiliations:** ^1^Laboratory of Viral Genomics, Pathogen Genomics Center, National Institute of Infectious DiseasesTokyo, Japan; ^2^Influenza Virus Research Center, National Institute of Infectious DiseasesTokyo, Japan; ^3^Research Center for Zoonosis Control, Hokkaido UniversityHokkaido, Japan

**Keywords:** MD simulation, influenza A(H3N2), HA protein, N-linked glycans, mutations, structural change

## Abstract

Influenza A(H3N2) has been a major cause of seasonal influenza in humans since 1968, and has evolved by antigenic drift under the constantly changing human herd immunity. Increasing evidence suggests that the antigenic change occasionally occurred concomitant with the alterations of the N-glycosylation site profile and hemagglutination activity of the virion surface protein hemagglutinin (HA). However, the structural basis of these changes remains largely unclear. To address this issue, we performed molecular dynamics simulations of the glycosylated HA trimers of the A(H3N2), which has a novel pattern of Asn-X-Ser/Thr sequons unique in the new A(H3N2) epidemic clade 3C.2a and is characterized by attenuated ability to agglutinate nonhuman erythrocytes. Comparison of the equilibrated structures of the glycosylated HA trimers with and without the 3C.2a-specific mutations reveals that the mutations could induce a drastic reduction in the apical space for the ligand binding via glycan-shield rearrangement. The results suggest that the 3C.2a strain has evolved an HA structure that is advantageous for evading pre-existing antibodies, while also increasing the ligand binding specificity. These findings have structural implications for our understanding of the phenotypic changes, evolution, and fate of influenza A(H3N2).

## Introduction

The hemagglutinin (HA) protein of influenza virus is a glycosylated type I integral membrane protein that protrudes from the mature virion surface and plays critical roles in viral interactions with hosts. The HA protein is synthesized in infected cells as a precursor HA0, and is subsequently cleaved by cellular proteases to HA1 and HA2 subunits that are covalently attached by a disulfide bond. The mature HA protein on the virion is composed of three pairs of the HA1/HA2 subunits (Ha et al., 2003). The tip of the HA protein forms a globular structure, termed the globular head, and confers on the virus an ability to attach cells via interactions with the sialic acid-containing glycan moiety on the target cell surface (Ha et al., 2003). Meanwhile, the HA globular head constitutes the major viral antigenic sites that induce neutralization antibodies in infected hosts. These functional and antigenic features drive sequence and structural variations, particularly near the receptor-binding site in the globular head, according to specific rules (Smith et al., 2004; Koel et al., 2013). Importantly, the sequence variation on the globular head causes various phenotypic changes of viruses, including changes in antigenicity and receptor specificity. Therefore, it is critical to determine the structural changes in the HA globular head in order to understand the viral interplay with the hosts and evolution. Unfortunately, however, it is usually time consuming to characterize mutation-induced structural changes by experimental approaches alone.

Computational science is a rapidly growing area that now successfully complements the experimental and theoretical sciences in various fields, including life science. For example, recent advances in molecular dynamics (MD) simulation enable us to characterize changes in the three-dimensional structures of the mutated proteins in relatively short timescales compared with the experimental approaches (Ode et al., 2012; Sato et al., 2013). The MD simulations have been used to disclose the structural basis of the adaptation and evolution of the highly mutable human immunodeficiency virus (HIV). This includes elucidation of the HIV structural changes associated with the phenotypic changes in viral neutralization sensitivity and receptor tropism (Naganawa et al., 2008; Yokoyama et al., 2012, 2016; Kuwata et al., 2013), viral sensitivity to antiviral protein (Miyamoto et al., 2012), viral drug sensitivity (Yuan et al., 2013), viral growth in non-natural host cells (Yokoyama et al., 2016), and viral sensitivities to antibodies by drug-resistance mutations (Alam et al., 2016; Hikichi et al., 2016).

In this study, we used the MD simulation to gain new insights into the roles of mutations in a recent epidemic variant of the influenza A(H3N2) viruses. The A(H3N2) viruses have emerged on 1968 in humans of southern Asia and were soon widespread in the world. Thereafter, the A(H3N2) has been a major cause of seasonal influenza in humans to date. During the 2014/15 epidemic season of influenza, a new A(H3N2) substrain had rapidly predominated in humans worldwide (Skowronski et al., 2016). Notably, the hemagglutination activity of this substrain somehow could be measured with only a small portion of the viral population using a conventional hemagglutination assay with nonhuman erythrocytes (Skowronski et al., 2016). The A(H3N2) substrain is characterized by alterations of the N-glycosylation sequons on the globular heads of the HA protein as compared with other A(H3N2) clades (Skowronski et al., 2016) and is now referred to as 3C.2a. The oligosaccharides on the HA protein play key roles in viral antigenicity (Aytay and Schulze, 1991; Abe et al., 2004; Saito et al., 2004; Ping et al., 2008; Das et al., 2010; Wang et al., 2010; Wanzeck et al., 2011) and binding specificity/affinity to the cellular receptor (Gunther et al., 1993; Ohuchi et al., 1997; Gambaryan et al., 1998; Matrosovich et al., 1999; Tsuchiya et al., 2002; Wang et al., 2009; de Vries et al., 2010; Liao et al., 2010). However, it remains unclear how the 3C.2a mutations altered the HA structure and attenuated the hemagglutination activity with nonhuman erythrocytes. To address this issue, we here examined the structural effects of the four mutations in the globular heads using MD simulations. The obtained results predicted that the mutations could induce rearrangement of the glycan shield around the receptor-binding surface of the HA protein, leading to shrinkage of the ligand-accessible space.

## Materials and Methods

### Genetic Clades Determination

Genetic clades determination of the influenza virus in Japan has been performed routinely as the part of the work of the National Epidemiological Surveillance of Infectious Diseases in Japan^1^ and the Global Surveillance of Influenza in the WHO Reference Laboratories^2^. Information on the specimen collection and weekly report of HA type is available on the National Epidemiological Surveillance of Infectious Diseases^3^. Briefly, RNAs were extracted from viruses by using QIAamp viral RNA kit (QIAGEN, Dusseldorf, Germany). The HA genes were amplified from extracted RNAs by RT-PCRs using gene-specific primers (the primer sequences are available upon request) and SuperScript III One-step RT-PCR system with Platinum Taq (Invitrogen, Carlsbad, CA, USA). Sequencing reactions were performed with BigDye terminator kit (Applied Biosystems) and sequences were determined using 3730xl DNA analyzer (Applied Biosystems, Foster City, CA, USA). The genetic clades were determined by the generation of phylogenetic trees of the HA genes. The phylogenetic trees were constructed using MEGA 6 software (Tamura et al., 2013) with the neighbor-joining method. The numbers of HA sequences obtained in the individual periods are 39, 83, 125, 108, 70, and 77 for the September 2013 – Jan. 2014, February 2014 – August 2014, September 2014 – January 2015, February 2015 – August 2015, September 2015 – January 2016, and February 2016 – August 2016, respectively. The nucleotide sequences used in this study are registered at GISAID, a publicly accessible influenza virus database^4^. Accession number at GISAID for the HA sequence used for the molecular modeling in this study is EPI543763 (A/Switzerland/9715293/2013 strain).

### Molecular Modeling of a Glycosylated HA Trimer in the Ligand-free State

Three-dimensional (3-D) models for glycosylated extracellular domains of HA trimers of influenza A (H3N2) in the ligand-free state were constructed by homology modeling with Molecular Operating Environment (MOE) (Chemical Computing Group Inc., Montreal, QC, Canada). The crystal structure of the HA trimer of the influenza A/Victoria/361/2011 (H3N2) virus (PDB code: 4O5N; resolution: 1.75 Å; amino acid residues 4–325 and 330–502 for HA1 and HA2 peptides, respectively) was used as the modeling template. Obtained models were optimized by energy minimization using MOE and an Amber10: Extended Huckel Theory (EHT) force field implemented in MOE, which combines Amber10 and EHT bonded parameters for the large-scale energy minimization (Gerber and Muller, 1995; Case et al., 2005). The high-mannose oligosaccharide Man_5_GlcNAc_2_ was added to potential *N*-glycosylation sites in HA using Online Glycoprotein Builder^5^.

### MD Simulation of Glycosylated HA Trimer Models

Glycosylated HA trimer models in a ligand-free state were subjected to MD simulation essentially as described for simulations of HIV-1 gp120 (Yokoyama et al., 2016). MD simulations were performed by the PMEMD (Particle Mesh Ewald Molecular Dynamics) module in the AMBER 14 program package (Case et al., 2014), employing the Amber ff99SB-ILDN force field, a protein force field with improved side-chain torsion potentials (Lindorff-Larsen et al., 2010), the GLYCAM06 force field, a biomolecular force field for glycans (Kirschner et al., 2008), and the TIP3P water model for simulations of aqueous solutions (Jorgensen et al., 1983). Bond lengths involving hydrogen were constrained with SHAKE, a constraint algorithm to satisfy a Newtonian motion (Ryckaert et al., 1977), and the time step for all MD simulations was set to 2 fs. A non-bonded cutoff of 10 Å was used. After heating calculations for 20 ps until 310 K using the NVT ensemble for the constant volume, temperature, and numbers of particles in the system, simulations were executed using the NPT ensemble for the constant pressure, temperature, and numbers of particles in the system at 1 atm, at 310 K, and in 150 mM NaCl for 100 ns. Root mean square deviations (RMSDs) between the heavy atoms of the two superposed proteins were used to measure the overall structural differences between the two proteins (Case et al., 2005). The RMSD was calculated using the cpptraj module in AmberTools 14, a trajectory analysis tool (Case et al., 2014). We used “Computer System for the Prediction of Mutations of Pathogens” at Research Center for Zoonosis Control, Hokkaido University for the MD simulations.

### Calculation of Root Mean Square Fluctuation (RMSF)

We calculated RMSFs of individual components of the high mannose oligosaccharides Man_5_GlcNAc_2_ around the receptor binding site between 50 to 100 ns of MD simulations to quantify structural dynamics of glycans during the MD simulations. The average structures during the last 50 ns of MD simulations were used as reference structures for RMSF calculation. RMSFs were calculated as previously described (Naganawa et al., 2008; Yokoyama et al., 2012, 2016; Kuwata et al., 2013) by using the ptraj module in Amber, a trajectory analysis tool (Case et al., 2005).

## Results

### Temporal Dynamics of Clade Populations of A(H3N2) Viruses Since September 2013

**FIGURE 1:**
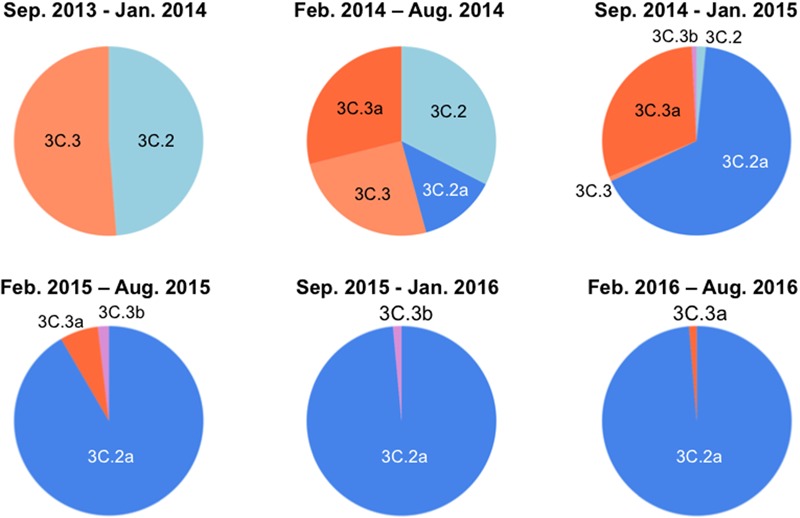
**Temporal dynamics of clade populations of A (H3N2) viruses since September 2013.** The clade-populations based on the phylogenetic tree of the hemagglutinin (HA) genes are shown by pie charts. The periods of each pie chart are determined by the collection dates of specimens. The clades are colored as follows: 3C.2, light blue; 3C.2a, blue; 3C.3, light orange; 3C.3a, orange; 3C.3b, light magenta.

Over the 2014/15 influenza season, influenza surveillance reports from reference laboratories warned of a global epidemic of a new A(H3N2) variant population, termed 3C.2a. In Japan, the 3C.2a was first detected as a relatively minor population over the season from February to August in 2014, during which four A(H3N2) clades co-existed (**Figure 1**, upper panel). However, the 3C.2a rapidly became dominant at the beginning of the winter season in 2015, displaced pre-existing clades, and has continually predominated at the collection sites in Japan ever since, representing 76 of 77 (98.7%) clades in the season from February to August in 2016 (**Figure 1**, lower panel). In parallel, another A (H3N2) clade, 3C.3a, which co-existed in the same period as the 3C. 2a over the February to August 2014 season, became minor after February 2015 (**Figure 1**). These results are consistent with the reports on the global epidemic of the 3C.2a from the WHO Reference Laboratories^6^, and suggest the selective advantage of the 3C.2a for human-to-human transmission during the study period. Notably, hemagglutination activity of the 3C.2a was significantly attenuated when measured with a conventional hemagglutination assay (Skowronski et al., 2016). Together, these data suggest that certain structural changes occurred in the HA protein to confer selective advantages on the 3C.2a.

### Amino Acid Signatures of the 3C.2a HA Protein

**FIGURE 2:**
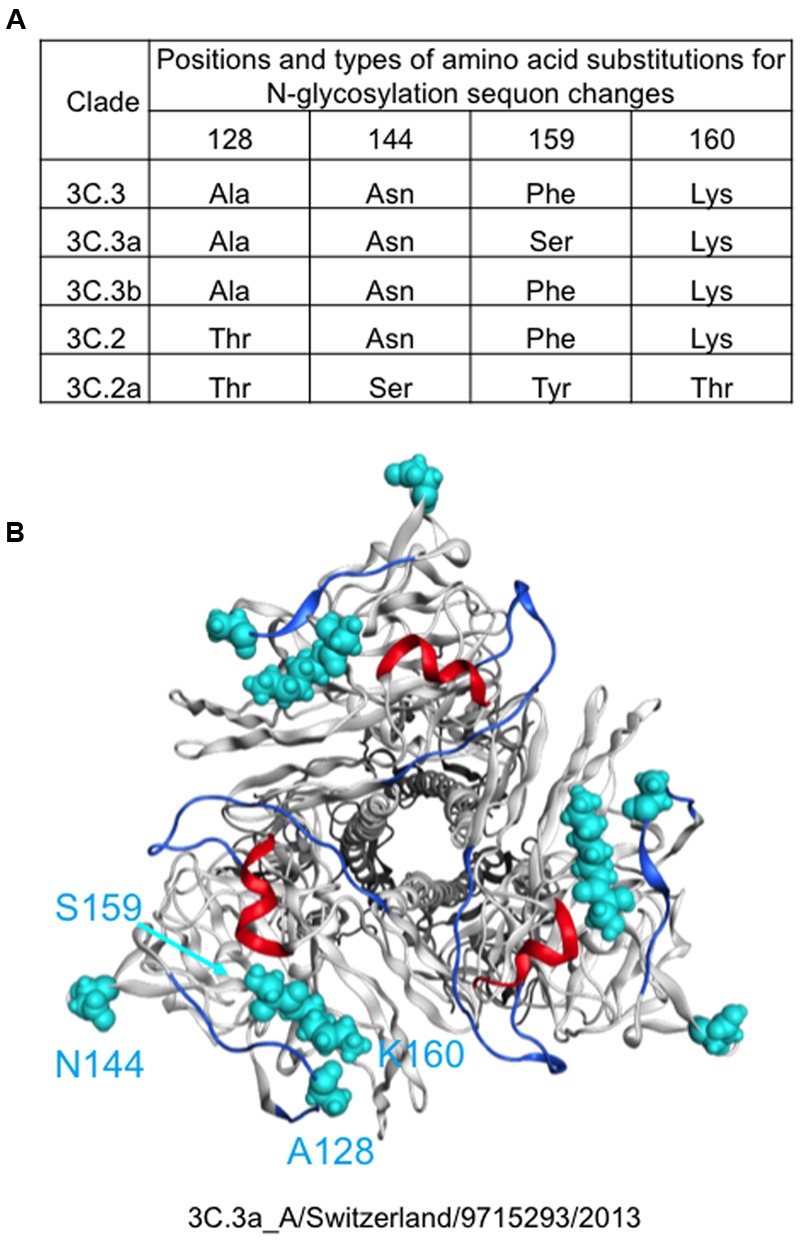
**Amino acid signatures of the 3C.2a HA protein.** Types and 3-D locations of amino acid substitutions around the receptor-binding surface on the tip of the HA protein are shown. **(A)** Types of amino acid residues among five A(H3N2) variant strains emerged in the 2013–2016 periods in Japan. **(B)** Locations of different amino acid residues between 3C.3a and 3C.2a are highlighted by cyan color on the HA protein model of the 3C.3a strain (A(H3N2) 2015/16 vaccine strain: A/Switzerland/9715293/2013).

Molecular epidemiological data from above study and the global surveillance of influenza in the WHO Reference Laboratories suggest that the A (H3N2) clades, 3C.2a and 3C.3a, were diversified from 3C.2 and 3C.3, respectively and thereafter the 3C.2a had dominated over the 3C.3a. Therefore, we examined differences in HA proteins between the clades 3C.2a and 3C.3a. The HA protein of the 3C.2a population has multiple amino acid substitutions as compared with the clade 3C.3a (amino acid numbers 3, 128, 138, 142, 144, 159, 160, 311, 326, and 489). In this study, we focused on the four substitutions around the receptor-binding surface on the tip of the HA protein, i.e., Ala128Thr, Asn144Ser, Ser159Tyr, and Lys160Thr (**Figure 2**). The Ala128Thr and Lys160Thr create new potential N-glycosylation sites, Asn-X-Ser/Thr, whereas Asn144Ser results in the loss of a single N-glycosylation site. Ser159Tyr is located adjacent to the Lys160Thr. The Ala128Thr substitution initially detected in the 3C.2 and had been preserved in its descendent, 3C.2a, whereas the other three substitutions newly emerged in the 3C.2a (**Figure 2A**). All the mutations are placed at or near the major antigenic elements for H3 HAs, such as antigen sites A (amino acids 140-146) and B (amino acids 155-160 and 188-198) (Webster and Laver, 1980; Gerhard et al., 1981; Wiley et al., 1981; Knossow et al., 1984; Wiley and Skehel, 1987) (**Figure 2B**).

### MD Simulations of Glycosylated HA Trimers in the Ligand-free State

To understand the structural impacts of the four mutations on the tip of the 3C.2a HA protein, we constructed molecular models of the glycosylated HA trimers with and without the mutations. Two structural models of the glycosylated HA trimers in a ligand-free state were constructed by homology modeling: a model for the A(H3N2) 2015/16 vaccine strain (A/Switzerland/9715293/2013) that belongs to the perishing clade 3C.3a (**Figures 1, 2**) and a model for A/Switzerland/9715293/2013 possessing the four 3C.2a-specific mutations on the HA globular heads (Ala128Thr, Asn144Ser, Ser159Tyr, and Lys160Thr). The obtained models were optimized by energy minimization using the MOE:EHT force field (Gerber and Muller, 1995; Case et al., 2005) and were subjected to the MD simulations using the Amber ff99SB-ILDN force field (Lindorff-Larsen et al., 2010) and the GLYCAM06 force field (Kirschner et al., 2008) as described previously (Yokoyama et al., 2016).

**FIGURE 3:**
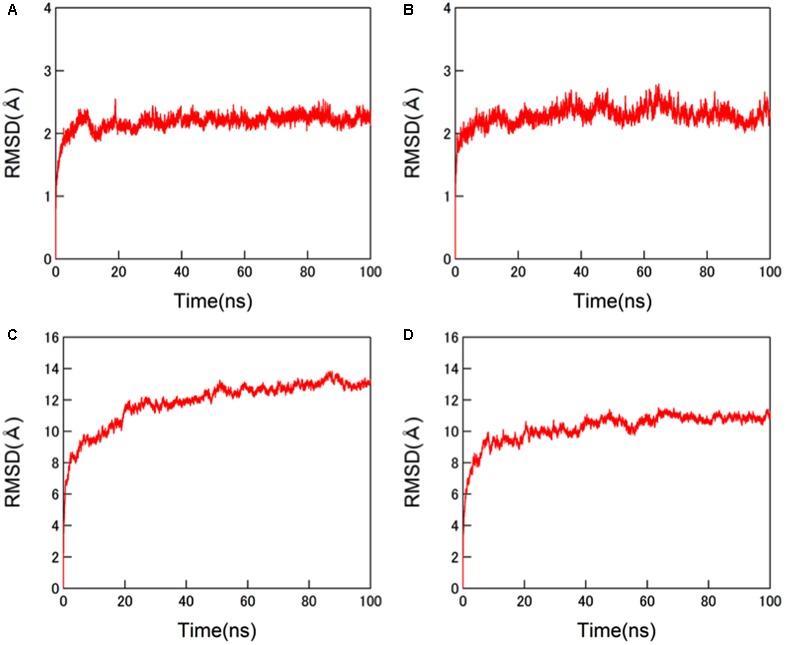
**Molecular dynamics (MD) simulations of glycosylated HA trimers in a ligand-free state.** Molecular models of glycosylated extracellular domains of HA trimer in a ligand-free state were constructed by homology modeling using MOE (Chemical Computing Group Inc., Montreal, QC, Canada) and subjected to MD simulations using the AMBER 14 program package (Case et al., 2014) as described in the Section “Materials and Methods”. The structural dynamics of the HA trimers in solution were monitored by root mean square deviations (RMSD) between the initial model structure and the structures at given time points of MD simulation. **(A,C)** HA trimer of a representative strain (A/Switzerland/9715293/2013) belonging to the subclade 3C.3a. **(B,D)** HA trimer of the A/Switzerland/9715293/2013 with Ala128Thr, Asn144Ser, Ser159Tyr, and Lys160Thr substitutions. **(A,B)** RMSDs of the protein portions. **(C,D)** RMSDs of N-glycans.

The structural dynamics of the glycosylated HA proteins in solution were monitored by RMSD between the initial model structure and the structures at given time points of the MD simulation (**Figure 3**). For the protein portions of the glycosylated HA molecules, the RMSDs sharply increased in the beginning and reached a near plateau after 20 ns of the MD simulations in both the 3C.3a and its mutant with 3C.2a mutations (**Figures 3A,B**). This RMSD profile was similar to that obtained with the glycosylated HIV-1 gp120 protein (Yokoyama et al., 2016). In addition, we monitored the RMSDs of the glycan portions of the glycosylated HA molecules. We obtained an RMSD profile that was similar to that of the protein portions: there was a sharp increase soon after the MD simulation onset followed by a near plateau after 20 ns (**Figures 3C,D**). These results suggest that the structural distortions of the amino acid residues and glycans of the initial models were relieved shortly after the start of MD simulation under thermodynamic driving forces in solution. The data also predict that the HA structure can reach a state of thermodynamic equilibrium in solution.

**FIGURE 4:**
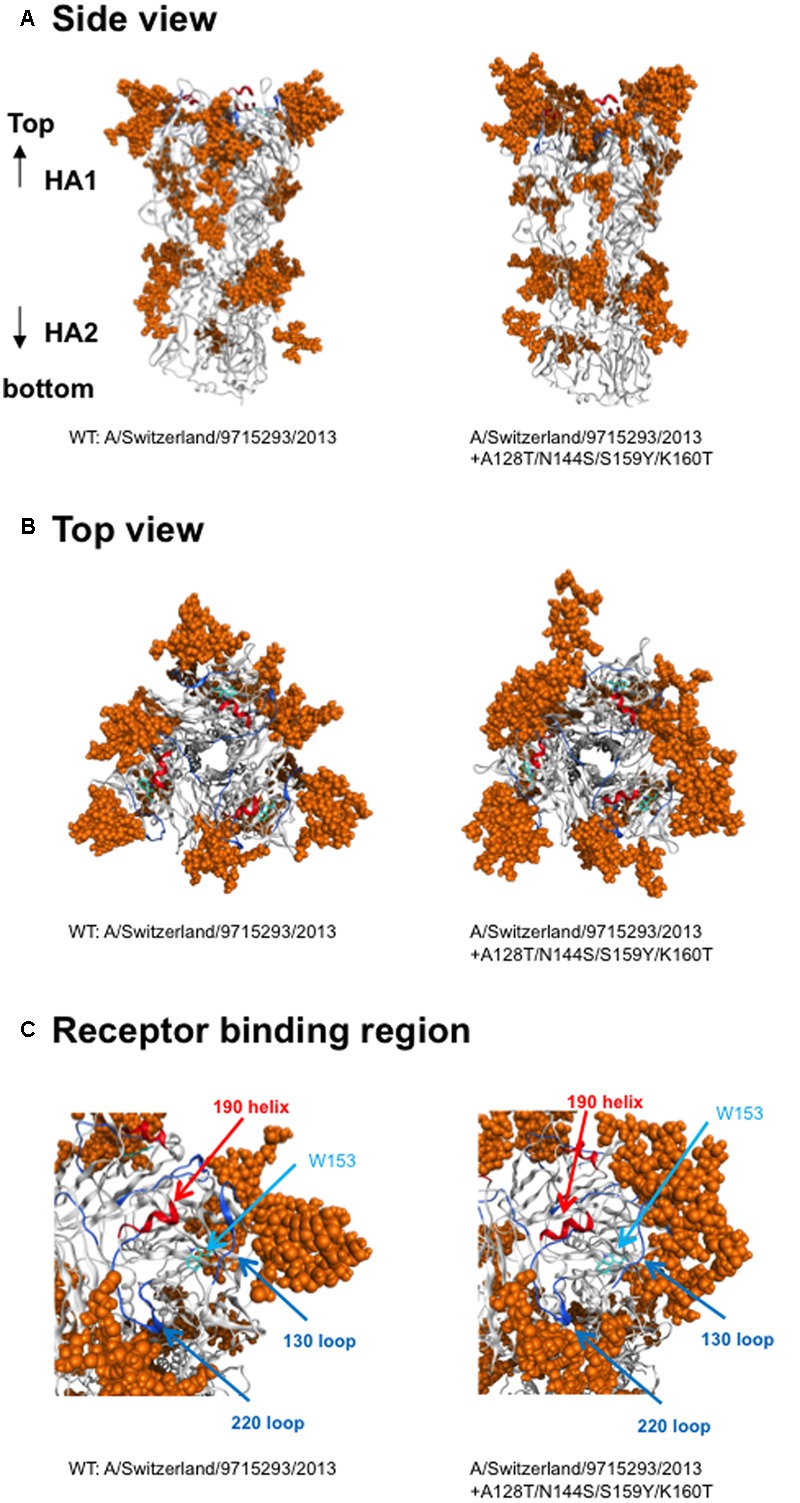
**Comparison of HA trimer models at 100 ns of MD simulations.** The 3-D structure of the HA trimer in a state of thermodynamic equilibrium during the MD simulations (100 ns) was compared between the vaccine strain and its mutant. **(A)** Side view, **(B)** top view. **(C)** Receptor-binding region. Helix and loops in the receptor-binding site are marked by red and blue colors, respectively. The tryptophan at position 153 for the binding of sialic acid is shown.

**FIGURE 5:**
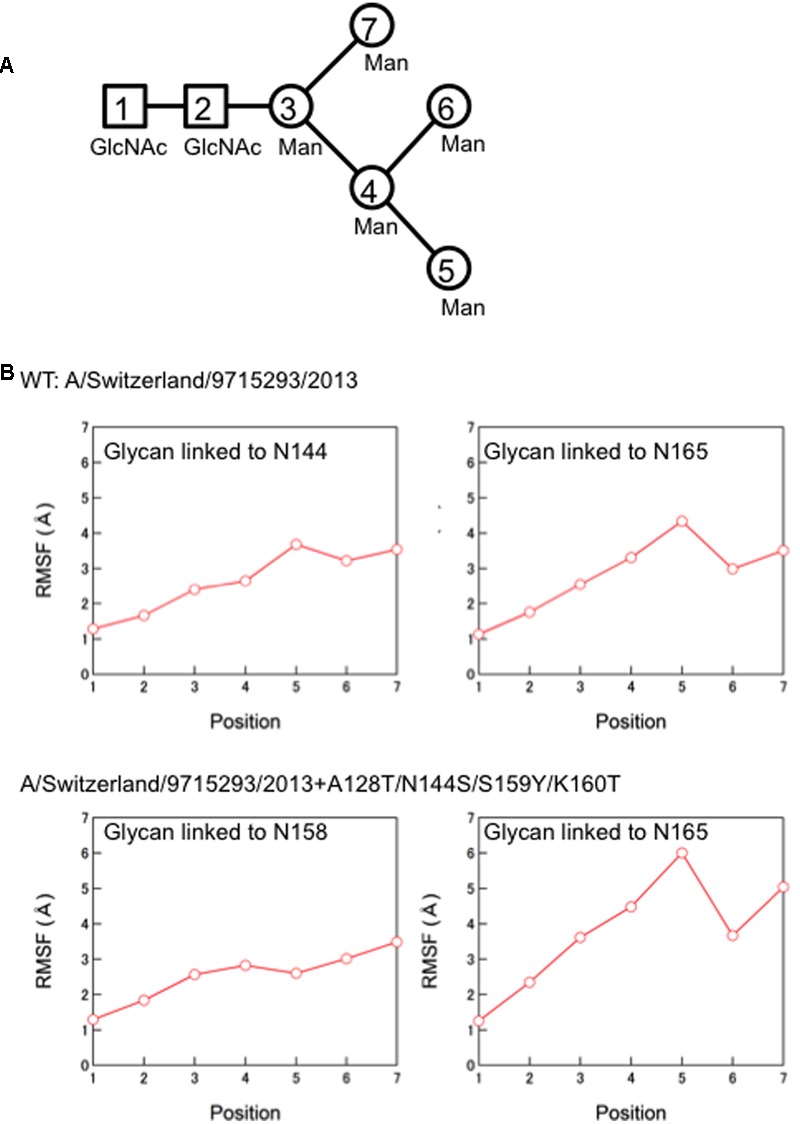
**Molecular dynamics of the individual glycans around the receptor binding site. (A)** Illustration of components of the high mannose oligosaccharides Man_5_GlcNAc_2_. **(B)** Root Mean Square Fluctuation (RMSF) of oligosaccharides. Structural fluctuations of the individual components of glycans around the receptor binding site of the HA proteins were analyzed by calculating RMSF using snapshots of structures from 50 to 100 ns of each MD simulation. The average structures during the last 50 ns of MD simulations were used as reference structures for RMSF calculation.

Finally, we compared the 3-D structure of the HA trimer in a state of thermodynamic equilibrium during the MD simulations (100 ns) between the 3C.3a and its mutant (**Figure 4A**). A marked structural difference between the 3C.3a and its mutants was detected on the globular heads of the HA protein at 100 ns of two MD simulations: the glycan moieties were placed more compactly in the mutant than in the 3C.3a (**Figures 4A,B**). Consequently, the space for the access of ligands around the receptor-binding site was more confined in the mutant (**Figure 4C**). The apical spaces for ligand binding could be influenced not only by the arrangements of glycans but also by fluctuation of the glycans. Therefore, we examined structural fluctuations of the individual glycans around the receptor binding site by calculating RMSF using snapshots of structures from 50 to 100 ns of each MD simulation. In the present HA models, the high mannose oligosaccharides Man_5_GlcNAc_2_ were attached on the asparagine residues of potential *N*-glycosylation sites around the receptor binding site (**Figure 5A**). We calculated RMSFs of individual components of the oligosaccharides, such as GlcNAc and Man, at positions 1 to 7 (**Figure 5B**). Notably, RMSFs of the glycan components at given positions, and the profiles of RMSFs at the seven positions were similar among the four *N*-glycosylation sites. The data indicate that all glycans fluctuated in a similar fashion around the receptor binding site during the MD simulations. These results suggest that the apical spaces for ligand binding are mainly influenced by arrangements of glycans and that the spaces are constantly different between the two models during the MD simulations.

## Discussion

In this report, we studied the structural impact of mutations residing in the HA globular head of a recent epidemic clade population of the A(H3N2). This clade, termed 3C.2a, predominated over other co-existing A(H3N2) clades including the clade 3C.3a during the 2014/15 and 2015/16 seasons, and retained a unique set of sequon mutations in the HA globular heads (**Figures 1, 2**). To analyze the HA structures under near physiological conditions, we performed MD simulations of the glycosylated HA trimers and obtained structures in a state of thermodynamic equilibrium in solution (**Figure 3**). Comparisons of the obtained structures revealed marked changes in the glycan shield around the receptor-binding site (**Figures 4, 5**). This finding has important implications for our understanding of phenotypic changes, evolution, and fate of the influenza virus A(H3N2).

First, the present results indicate that the 3C.2a-specific mutations have an impact on the immunological features of the HA protein. Our MD simulations show that the glycans on the HA globular head are rearranged by the mutations into a configuration markedly distinct from that of the 2015/16 vaccine strain (**Figures 4, 5**). Therefore, these mutations could induce changes in the HA antigenicity. These structural findings are consistent with previous reports demonstrating critical roles of the oligosaccharides on the HA protein in the viral antigenicity of influenza viruses (Aytay and Schulze, 1991; Abe et al., 2004; Saito et al., 2004; Ping et al., 2008; Das et al., 2010; Wang et al., 2010; Wanzeck et al., 2011). Moreover, we found that the glycan rearrangement resulted in shrinkage of the access space on the top of the HA protein near the receptor-binding site of the globular head (**Figure 4**). This structural change could cause steric hindrance for binding of the antibodies directed to the antigen site B. Thus, it is likely that the HA protein of the 3C.2a had a selective advantage in evading antibodies against receptor-binding site as compared with the 3C.3a HA. These possibilities are consistent with the rapid spread and predominance of the clade C3.2a over the clade 3C.3a in human populations during the study period (**Figure 1**) (Skowronski et al., 2016). Antigenic analysis of these viruses should be done to make sure these possibilities.

Secondly, the present results indicate that the 3C.2a-specific mutations impact the receptor specificity of the HA protein. Our MD simulations showed that the new arrangement of glycans on the HA globular head could shrink the space for the access of the sialic acid-containing glycan moiety on the target cell surface (**Figure 4**). This structural change could cause an increase in the ligand specificity and/or affinity of the HA protein, and thereby lead to a preference for receptors on the human respiratory organs, but not for receptors on the nonhuman erythrocytes. These structural findings are consistent with the attenuation of hemagglutination activity of the 3C.2a HA protein when assessed with a conventional hemagglutination assay using nonhuman erythrocytes (Skowronski et al., 2016). Similarly, previous studies have demonstrated that the oligosaccharides on the HA protein surface play key roles in the binding specificity and the affinity to infection receptors (Gunther et al., 1993; Ohuchi et al., 1997; Gambaryan et al., 1998; Matrosovich et al., 1999; Tsuchiya et al., 2002; Wang et al., 2009; de Vries et al., 2010; Liao et al., 2010).

Thirdly, the present findings have implications in terms of the adaptive evolution of A (H3N2). Previous reports have highlighted the importance of changes in the glycan shield for viral adaptation (Deom et al., 1986). Interestingly, the numbers of potential N-glycosylation sites in the HA protein have been continuously increasing since 1968: only two sequons existed –in the initial strains, whereas more than 7–10 sequons are common in the present epidemic strains (Blackburne et al., 2008). This change is likely to be a basic strategy by which A (H3N2) has maintained its presence in human populations with changing herd immunity over the last 48 years. However, the change also runs the risk of creating an evolutionary “dead-end” for the virus, because the acquisition of new glycans on the HA globular head increases the chances for steric hindrance during receptor binding. Notably, both the present and recent studies suggest that the HA protein possessing the 3C.2a-type glycan shield would significantly affect ligand specificity (**Figure 4**) (Skowronski et al., 2016). Thus, the 3C.2a has evolved an HA structure that is advantageous for evading pre-existing antibodies, while also reducing ligand affinity to nonhuman glycan moieties.

Finally, the present have structural implications in the fate of influenza A (H3N2) viruses. Our study indicates that an increase in the number of the glycan moieties around the receptor-binding site could induce a drastic reduction in the apical space for ligand binding (**Figure 4**). As discussed above, continuous interactions between A (H3N2) and human immunity seem to force the virus to serially increase the numbers of glycans on the HA protein. This eventually may reduce the HA binding affinity via an increase in steric hindrance even to the human ligands, and thereby decrease the replication fitness of the virus for spread among humans. Thus, if the overall tendency of HA to increase the numbers of glycosylation sites (Blackburne et al., 2008) continues in the future, it may eventually put the influenza A (H3N2) viruses in an evolutionary cul-de-sac.

## Author Contributions

MY, SW, TO, and HS conceived and designed the study. SF and MS performed sequencing and phylogenetic analysis. KI and HS prepared the computing environment. MY performed MD simulations. HS prepared the manuscript. All authors read and approved the final manuscript.

## Conflict of Interest Statement

The authors declare that the research was conducted in the absence of any commercial or financial relationships that could be construed as a potential conflict of interest.

## Footnotes

1 http://www.nih.go.jp/niid/en/influenza-e.html

2 http://www.who.int/influenza/en/

3 http://www.nih.go.jp/niid/en/influenza-e/2099-idsc/iasr-flu-e/6791-iasr-inf-e20160925.html

4 http://platform.gisaid.org/epi3/frontend

5 http://glycam.org/

6 http://www.who.int/influenza/en/
